# Real-world study on clinical outcomes of nucleos(t)ide analogues antiviral therapy in patients with chronic hepatitis B

**DOI:** 10.1017/S0950268819000815

**Published:** 2019-05-09

**Authors:** Y. Sun, Y. Zhang, Y. Xu, M. Shu, K. Bonroy, H. Qiu, W. Cai

**Affiliations:** 1 Department of Infectious Diseases, Ruijin Hospital, Shanghai Jiao Tong University School of Medicine, China; 2 Epidemiology, Janssen Research and Development, USA; 3 Janssen Pharmaceutica NV, Belgium

**Keywords:** Chronic hepatitis B, cirrhosis, compliance, hepatocellular carcinoma, nucleos(t)ide analogues

## Abstract

Nucleos(t)ide analogues (NAs) are widely used for antiviral therapy in patients with chronic hepatitis B (CHB), but real-world data on treatment patterns and long-term clinical outcomes are not always available. Using data from electronic medical records between January 2011 and December 2016 in Shanghai, China, we evaluated patient characteristics, treatment patterns and clinical outcomes in patients with CHB. There were 6688 patients in the study cohort. The incidences of cirrhosis and hepatocellular carcinoma (HCC) were 41.0‰ and 6.8‰ person-years, respectively. There were more cirrhosis and HCC cases among patients who had shorter NA treatment duration (<365 days), or who were less compliant (<80%). In addition, increased risk of cirrhosis and HCC was observed in patients who did not achieve hepatitis B surface antigen (HBsAg) loss/seroconversion. Moreover, patients with cirrhosis developed after antiviral treatments had a higher incidence of HCC (adjusted hazard ratio 15.86, 95% confidence interval 7.35–34.24). Good compliance with treatment and longer treatment duration significantly decreased the risk of developing cirrhosis and HCC. HBsAg loss seemed to be a protective factor for cirrhosis/HCC in NAs-treated patients with CHB, and cirrhosis was a confirmed risk factor for HCC development as expected.

## Introduction

Viral hepatitis is a leading cause of death worldwide, and more than half of the viral hepatitis deaths in China are caused by hepatitis B virus (HBV) [1]. It is well known that chronic HBV infection results in continuous liver injury and sometimes life-threatening complications, especially cirrhosis and hepatocellular carcinoma (HCC) [2, 3]. With an estimated 257 million people infected with HBV globally, 2–10% of these patients will progress to cirrhosis, and 0.2–0.6% of them will eventually develop HCC [4, 5]. The wide availability of antiviral drugs has significantly decreased the incidence rate of HBV-related cirrhosis and HCC [6–9]. However, liver disease progression and HCC development in patients with chronic hepatitis B (CHB) cannot be eliminated completely even in patients taking long-term nucleos(t)ide analogue (NA) therapy [9–11]. So, monitoring of disease progression in CHB patients taking antiviral therapy is still critical.

The development of cirrhosis and HCC in patients with CHB is complicated, dependent on numerous related factors including the host, virus and their interactions [12, 13]. Older age and male gender are both strongly associated with the development of cirrhosis and HCC [5]. Some studies demonstrate that elevated HBV DNA level is an independent risk factor for cirrhosis and HCC in CHB patients receiving no NA therapy [14–16]. High level of hepatitis B surface antigen (HBsAg) at diagnosis is also a risk factor for progression to HCC [16].

In this retrospective hospital-based, long-term study, we followed up a total of 6688 individuals with NA-treated CHB patients who had clinical records in the hospital information system (HIS). After starting NA treatment, levels of alanine aminotransferase (ALT), HBV DNA, HBsAg, hepatitis B surface antibody (HBsAb), hepatitis B e antigen (HBeAg) and hepatitis B e antibody (HBeAb) were consistently recorded in these patients. The goal of the study was to describe the ‘real-world’ antiviral NA treatment patterns, to evaluate treatment responses and to assess possible factors that were related to the development of cirrhosis and HCC in a CHB cohort receiving NA therapy.

## Methods

### Data source

The study was conducted in the Department of Infectious Diseases, Ruijin Hospital, Shanghai Jiao Tong University School of Medicine, which is a tertiary general hospital with more than 2000 beds in a first-tier city in China. It provides integrated healthcare services and represents one of the highest standards of care for patients with CHB in China. All physicians in the department are specialists in infectious disease and provide professional care for this disease area. The hospital established the HIS more than 18 years ago. Patients' data from various departments are linked through the unique patient identifier and are all stored in the HIS. Major clinical information, including patient demographics (birth year/month, gender, residency), visit dates, outpatient diagnoses, inpatient admission and discharge diagnoses, inpatient and outpatient treatment, procedures, laboratory test results and other diagnostic tests have been de-identified and transferred from the HIS into the research database. Diagnoses in the HIS database are coded using the International Classification of Diseases 10th revision (ICD-10).

### Study design

This was a retrospective cohort database study to describe patient characteristics and disease consequences among patients with CHB who received antiviral treatment in the Department of Infectious Diseases, Ruijin Hospital, Shanghai Jiao Tong University School of Medicine, China. Included patients were followed up from the earliest antiviral treatment date until the last visit date in the database. All clinical records of these patients, including data available back to the year 2000, were identified and extracted from the HIS. Antiviral medications extracted from prescription claims included lamivudine, adefovir, telbivudine and entecavir as mono- or combined therapies. This study was approved by the local Human Ethics Committee.

### Study population

Patients aged at least 18 years who had a diagnosis of CHB in the Department of Infectious Diseases from 1 January 2011 to 31 December 2016, who received continuous care (at least three consecutive visits in the outpatient clinic or hospitalisation with no gap longer than 90 days between visits) and NA antiviral treatment, were included in this study. Exclusion criteria were as follows: at least with one diagnosis record of hepatitis C, hepatitis D, hepatitis G or human immunodeficiency virus (HIV), or pre-existing liver cirrhosis or HCC in the database before the earliest antiviral treatment date. All patients had at least one HBV-related laboratory test during the study period.

### Laboratory tests

Laboratory test results for ALT, HBsAg, HBsAb, HBeAg, HBeAb and HBV DNA were captured from the HIS during the follow-up period. Serum HBV DNA quantification was determined using Applied Biosystems Real-time PCR system (Prism 7500; Applied Biosystems, Inc., USA, with a low limit of 500 IU/ml) or Roche COBAS HBV Amplicor MonitorTM assay (Roche Diagnostics, Basel, Switzerland; with a low limit of 20 IU/mL). Serum HBsAg, HBsAb, HBeAg and HBeAb were determined using commercial enzyme immunoassay kits (AXSYM System; Abbott, Wiesbaden, Germany). HBsAg was quantified by Abbott Architect HBsAg Reagent Kit (Abbott Ireland Diagnostics Division, Sligo, Ireland; dynamic range 0.05–250.0 IU/ml).

### Definition of events

NA treatment duration and treatment compliance of patients were determined. Treatment duration was the time from the treatment index date until a treatment gap occurred (more than 121 days without NA) or the end of the study, whichever is earlier. Treatment compliance is the total time on drug within 365 days post the treatment index date divided by the total time in the database in the first 365 days post the treatment index date.

Patient serological indices and diagnosis, including biological breakthrough (BBT), virological breakthrough (VBT), HBsAg loss or seroconversion, HBeAg loss or seroconversion, functional cure, as well as cirrhosis and HCC, were captured during the cohort follow-up. BBT was defined as detecting ALT >5 times upper limit of normal in patients whose ALT had been normalised, or ALT >5 times nadir in those whose ALT level was continuously abnormal. VBT was defined as any increase in HBV DNA by >1 log_10_ from nadir, or ⩾10-fold the lower limit of detection of the HBV DNA assay. Seroconversion of HBsAg or HBeAg was defined as the development of HBsAb or HBeAb positivity in addition to loss of antigen, respectively. Functional cure was defined as loss of HBsAg with maintained virological remission (HBV DNA <2000 IU/ml) and ALT normalisation.

### Statistical analysis

The person-years of follow-up were calculated from the date of the earliest NA treatment until the date of the relevant event (cirrhosis or HCC), the last visit date or the end date of study (31 December 2016), whichever was earliest. Incidence rates were calculated as the number of new cases divided by person-years of follow-up. When analysing the effect of serological changes on the incidence of cirrhosis or HCC, occurrence of cirrhosis or HCC within 6 months of serological change was not counted, in order to exclude those pre-existing cases with delayed diagnosis. For the same reason, in the patients who did not have serological change, the occurrence of cirrhosis or HCC within 6 months from initial NA treatment was also excluded. Cox proportional hazard regression models were utilised in comparative analyses and hazard ratios (HR) and their 95% confidence intervals (CI) were estimated. Age and gender at baseline were considered as potential confounders and have been adjusted for in the Cox proportional hazard regression model. All analyses were conducted using SAS version 9.4 (Cary, NC, USA).

## Results

**Fig. 1.**
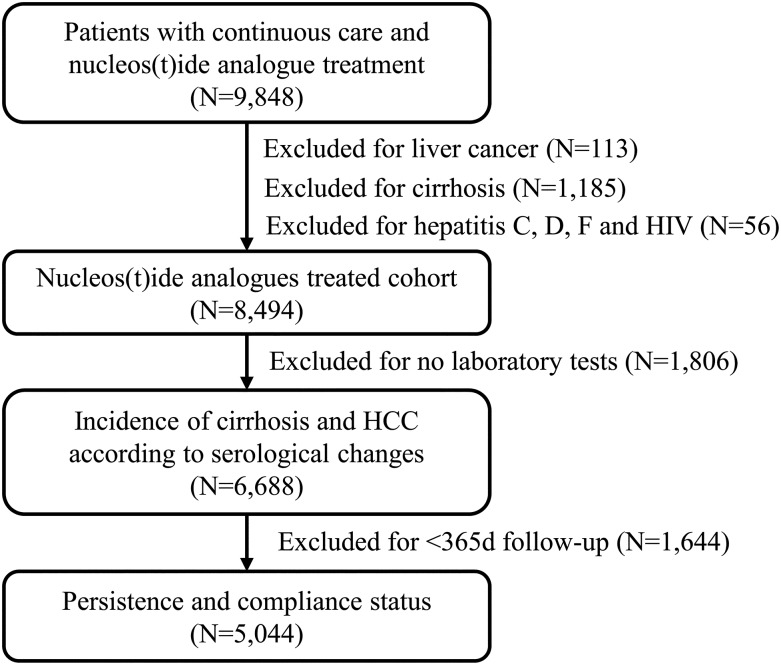
Flow chart of the study cohort

There were 9848 patients with CHB infection identified from the HIS who received NA treatment, continuous care and met eligibility criteria (Fig. 1). There were 113 patients who were excluded since they have been diagnosed with HCC before the date of the first NA treatment, 1185 had cirrhosis and 56 patients had been diagnosed with co-infections with hepatitis C, hepatitis D, hepatitis G or HIV. Of the remaining 8494 patients, there were 6688 patients who had at least one HBV-related laboratory test available for the main analysis.

### Baseline demographic characteristics

The average follow-up time of the 6688 patients included in the study was 2.2 years (standard deviation (s.d.) 1.3 years). Characteristics of the CHB patients at our centre are shown in Table 1. More than half of the patients were male (*n* = 4422, 66.1%) and the age of the patients ranged from 18 to 97 years, with most between 30 and 39 years of age (*n* = 1912, 28.6%). A total of 3021 patients (45.2%) were local residents in Shanghai. Hypertension and diabetes were the most common comorbidities at cohort entry. The mean duration of NA therapy was 416.2 days (s.d. 437.0) (Table 2). The most common initial treatment was entecavir monotherapy (55.0%), followed by lamivudine monotherapy (16.0%) and telbivudine monotherapy (14.6%). Adefovir monotherapy (5.4%) was uncommon and only 9.1% of patients received combined therapy. Patients had a mean of 27.9 (s.d. 29.4) healthcare visits at the hospital (inpatient or outpatient for any reason) during the study (Table 2).

**Table 1.** Demographic and clinical characteristics at study entry in the chronic hepatitis B cohort study

Characteristics	Patients with chronic hepatitis B (*n* = 6688)
Age (years)	
Mean (s.d.)	43.2 (14.6)
18–29 (n)	1296
30–39	1912
40–49	1186
50–59	1260
60–69	747
⩾70	287
Gender, n (%)	
Male	4422 (66.1)
Female	2266 (33.9)
Province (%)	
Shanghai	3021 (45.2)
Zhejiang	678 (10.1)
Anhui	549 (8.2)
Jiangsu	539 (8.1)
Jiangxi	421 (6.3)
Others	1480 (22.1)
Major coexisting diseases (%)	
Hypertension	240 (3.6)
Diabetes	168 (2.5)
Chronic gastritis	108 (1.6)
Fatty liver disease	90 (1.4)
Chronic nephritis	78 (1.2)

s.d., standard deviation.

**Table 2.** Treatment characteristics in the chronic hepatitis B cohort, 2010–2016

Variable	2010–2016 (*n* = 6688)
First prescription antiviral drug, n (%)	
Lamivudine	1068 (16.0%)
Adefovir	358 (5.4%)
Telbivudine	978 (14.6%)
Entecavir	3678 (55.0%)
Combined antiviral drugs	606 (9.0%)
Antiviral therapy duration, days	
Mean (s.d.)	416.2 (437.0)
Median (min, max)	229 (2–1530)
Visits, n	
Mean (s.d.)	27.9 (29.4)
Median (min, max)	16 (3–594)

s.d., standard deviation.

### Changes in the distribution of NA agents

**Fig. 2.**
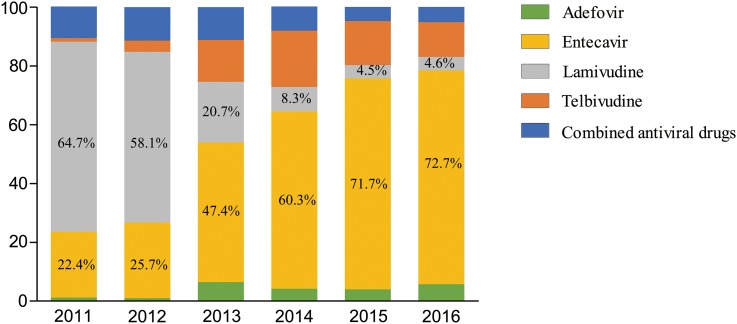
Percentage drug distribution in patients with nucleos(t)ide analogues from year 2011 to year 2016

With the admission of new drugs into the Chinese market, initial NA therapy for CHB patients under care in the hospital changed significantly over the study period. Lamivudine was the first antiviral drug available in the Chinese market, but gradually with the appearance of NA drugs which were more potent and had higher resistance barrier, lamivudine became a second-line drug in the clinical guidelines. As a result, lamivudine prescribing dropped 41% between 2011 and 2016 (64.7% in 2011 *vs.* 4.6% in 2016) annually. On the other hand, entecavir was the only first-line NA antiviral drug in the Chinese market until 2016. In our study, we observed a parallel increase in entecavir prescriptions from 22.4% in 2011 to 72.7% in 2016. Additionally, prescriptions of combined antiviral NA therapy decreased in CHB patients over these years (Fig. 2).

### Associations between treatment patterns and progression to advanced liver diseases

In our study cohort, there were 1644 patients with <365 days of follow-up and these patients were excluded from the analysis of the effect of treatment duration and compliance on the incidence of cirrhosis or HCC (Fig. 1). Of the rest patients, 2582/5044 (51.2%) had good treatment duration (i.e. received NA treatment for at least 365 days), and 2800 (55.5%) were compliant with treatment (i.e. ⩾80% compliant with NA treatment in the first year of the follow-up period) (Table 3). The incidence of cirrhosis and HCC was significantly higher in patients with shorter treatment duration (adjusted HR 1.42, 95% CI 1.18–1.71 for cirrhosis and adjusted HR 1.96, 95% CI 1.23–3.13 for HCC). Similarly, the incidences of cirrhosis and HCC were also significantly higher in patients who were not compliant with treatment (adjusted HR 1.49, 95% CI 1.24–1.79 for cirrhosis and adjusted HR 2.12, 95% CI 1.33–3.38 for HCC).

**Table 3.** Incidence rates of cirrhosis and hepatocellular carcinoma in antiviral CHB cohort by treatment duration and compliance

	No. of patients	Cases	Person-years of follow-up	Incidence rate (per 1000 person-years)	Crude HR (95%CI)	Adjusted HR^a^ (95%CI)
Duration						
Cirrhosis						
<365 days	2462	236	5571.7	42.4	1.26 (1.04–1.51)	1.42 (1.18–1.71)
⩾365 days	2582	219	7255.3	30.2		
HCC						
<365 days	2462	43	5991.4	7.2	1.64 (1.03–2.61)	1.96 (1.23–3.13)
⩾365 days	2582	32	7643.4	4.2		
Compliance						
Cirrhosis						
<80%	2244	224	5131.9	43.7	1.33 (1.10–1.60)	1.49 (1.24–1.79)
⩾80%	2800	231	7695.2	30.0		
HCC						
<80%	2244	42	5528.4	7.6	1.80 (1.13–2.86)	2.12 (1.33–3.38)
⩾80%	2800	33	8106.3	4.1		

a Adjusted for age and gender. *P*-value = *χ*^2^ test.

HR, hazard ratio; CI, confidence interval; HCC, hepatocellular carcinoma.

### Associations between serological changes and progression to cirrhosis

There were 562 patients diagnosed with cirrhosis during follow-up in the NAs CHB cohort (incidence 41.0 per 1000 person-years). However, only patients with cirrhosis diagnosed at least 183 days after the serological change were considered as outcomes for the analysis in Table 4. None of the 53 patients with HBsAg loss and 26 patients with HBsAg seroconversion, or the 50 patients with a functional cure was diagnosed with cirrhosis during the study period. No significant associations were identified between BBT, VBT, HBeAg loss/seroconversion and the occurrence of cirrhosis.

**Table 4.** Number and incidence of patients who developed chronic hepatitis B related cirrhosis, diagnosed at least 183 days after the serological event

Varieties	No. of patients^a^	Cirrhosis cases	Person-years of follow-up	Incident rate (per 1000 person-years)	Adjusted HR^b^ (95%CI)
Total	6688				
BBT					
Yes	47	1	70.6	14.2	0.97 (0.14–6.96)
No	4489	189	11 063.3	17.1	
VBT					
Yes	346	6	640.5	9.4	0.60 (0.27–1.37)
No	4459	182	10 501.0	17.3	
HBeAg loss					
Yes	388	7	787.0	8.9	
No	1403	37	3281.9	11.3	1.32 (0.59–2.97)
HBeAg seroconversion					
Yes	224	2	453.6	4.4	
No	1468	42	3539.9	11.9	2.42 (0.58–10.02)
HBsAg loss					
Yes	53	0	90.6	0.0	
No	3387	140	8598.93	16.3	−
HBsAg seroconversion					
Yes	26	0	40.1	0	
No	3210	133	8147.3	16.3	−
Functional cure					
Yes	50	0	86.7	0	−
No	4917	209	11 762.9	17.8	

a Patients with cirrhosis diagnosed in the first 183 days (6 months) after the serological event were not counted.

b Adjusted for age and gender.

HR, hazard ratio; CI, confidence interval; BBT, biological breakthrough; VBT, virological breakthrough; HBeAg, hepatitis B e antigen; HBsAg, hepatitis B surface antigen.

### Associations between serological changes and progression to HCC

There were 99 patients diagnosed with HCC during the study follow-up period (incidence of 6.8 per 1000 person-years). Possible risk factors that could affect HCC development were explored in patients diagnosed with HCC at least 183 days after the serological change (Table 5). In our study, cirrhosis was identified as the highest risk factor for HCC (adjusted HR 15.86, 95% CI 7.35–34.24). There were no cases of HCC among all 54 patients with HBsAg loss, none among all 27 patients with HBsAg seroconversion and none among all 51 patients who experienced a functional cure. However, there were no significant associations between BBT, VBT, HBeAg loss/seroconversion and the occurrence of HCC.

**Table 5.** Number and incidence of patients who developed hepatocellular carcinoma, diagnosed at least 183 days after the serological event

Varieties	No. of patients^a^	HCC cases	Person-years of follow-up	Incident rate (per 1000 person-years)	Adjusted HR^b^ (95%CI)
Total	6688				–
BBT					
Yes	52	1	84.3	11.9	4.73 (0.64–34.87)
No	4722	40	11 862.8	3.4	
VBT					
Yes	383	4	87	5.6	2.09 (0.74–5.93)
No	4672	37	6480	3.3	
HBeAg loss					
Yes	411	2	840.2	2.4	
No	1447	8	3422.5	2.3	1.06 (0.22–5.04)
HBeAg seroconversion					
Yes	234	1	470.7	2.1	
No	1512	8	3694.5	2.2	0.97 (0.12–7.78)
HBsAg loss					
Yes	54	0	93.4	0	
No	3529	30	9123.4	3.3	–
HBsAg seroconversion					
Yes	27	0	42.9	0	
No	3339	28	8635.8	3.2	–
Functional cure					
Yes	51	0	89.4	0	
No	5161	50	12 599.2	4.0	–
Cirrhosis					
Yes	409	16	845.1	18.9	15.86 (7.35–34.24)
No	5443	12	13 090.3	0.9	

a Patients with HCC diagnosed in the first 183 days (6 months) after the serological events were not counted.

b Adjusted for age and gender.

HCC, hepatocellular carcinoma; HR, hazard ratio; CI, confidence interval; BBT, biological breakthrough; VBT, virological breakthrough; HBeAg, hepatitis B e antigen; HBsAg, hepatitis B surface antigen.

## Discussion

This retrospective study comprehensively describes current antiviral treatment duration and compliance of CHB patients as well as possible serological factors which may correlate with the development of cirrhosis and HCC. The large sample size and longitudinal follow-up of the study cohort enabled the analysis of clinical outcomes that usually require years to develop, which gave us much more insights of what NA treatment can achieve in a real-world setting in those patients with CHB. In the current study, treatment duration and treatment compliance to antiviral NA drugs significantly affected the development of cirrhosis and HCC in patients with CHB. In addition, HBsAg loss may be a protective factor for developing cirrhosis or HCC. As expected, cirrhosis was still a significant risk factor for HCC development even in NA-treated patients.

Based on clinical practice and experience, entecavir and tenofovir were recommended as the first-line antiviral drugs for CHB in the guideline [17], which owned to their good efficacy and high resistance barrier. Changes of trend in NA treatment over years in our study were consistent with the changes in the guideline and reflect the changes in the management of patients with CHB in the Chinese population.

Current guidelines suggest continuous NA treatment, in patients with active CHB disease. Therapies that require continuous treatment are expected to result in poor compliance and some patients even giving up on treatment. Poor compliance is reported to be associated with an increased risk of virological failure [18]. In our real-world study, we showed that treatment duration and compliance had a significant effect on CHB outcomes during the follow-up period, with a lower risk of developing cirrhosis and HCC among patients who showed good compliance with therapy and longer treatment duration. It suggested that patients with antiviral therapy should be monitored regularly for a better outcome. However, patients with >365 days treatment duration in this study had a much longer follow-up period than the patients with <365 days treatment duration. Patients with longer treatment duration probably preferred receiving various healthcare services in the same hospital, which led to a longer follow-up period. When calculating the incidence rates, both person-years and number of cases may be overestimated simultaneously, which might cause potential minor bias.

Underlying liver diseases, including chronic viral hepatitis, can contribute to the development of liver cirrhosis [19]. Although not all patients with cirrhosis are certain to develop HCC, there are strong links between the two pathologies [20]. This has been described for patients with CHB in whom cirrhosis can predict the incidence of HCC [21], and in a study that showed that in the absence of antiviral therapy, the rate of HCC was 22-fold higher among CHB patients with cirrhosis compared to those without [22]. Sub-classification of cirrhosis even could be used as a significant predictor of late recurrence in patients with HBV-related HCC after surgery [23]. Our study confirms an important role of cirrhosis in HCC development; cirrhosis was associated with over a 15-fold evaluated risk to develop HCC in patients receiving antiviral therapy.

In patients with CHB, the rate of HBsAg loss ranges from 0.0% to 3.0% annually with entecavir or tenofovir treatment [24], and an HBsAg loss rate of 8.4‰ annually was observed in our NA-treated CHB cohort. HCC can develop even after HBsAg loss in the absence of antiviral treatment [25]. However, no cases of HCC after HBsAg loss during antiviral therapy were observed in our real-world retrospective study, even no cirrhosis cases occurred. Our findings were similar with the Korean study, which shows a lower incidence of HCC in patients with HBsAg loss compared to patients without HBsAg loss for patients with CHB taking antiviral therapy over 6 years [26]. Our further study will be researched with a larger number of cases in the future, in order to provide strong evidence to illustrate the role of HBsAg loss or seroconversion in HCC or cirrhosis.

The predictive value of HBeAg as a marker of disease progression in patients with CHB is also controversial. The Kawerau township study in Maori patients found that HBeAg positivity was predictive neither for liver cirrhosis, nor HCC[27], while conversely, a study in Taiwan showed that HBeAg positivity could be considered as a risk factor for HCC [28]. In another study of antiviral-treated patients with CHB, no difference in the HCC incidence rate was reported between HBeAg-positive and -negative patients [29]. Consistent with these observations, our study showed that HBeAg loss or seroconversion did not prevent the development of cirrhosis or HCC. No association was found between HBeAg loss and HCC, which may be due to our relatively small HCC cases. Accordingly, the role of HBeAg loss or seroconversion in the development of cirrhosis and HCC in CHB patients with antiviral treatment remains ambiguous. Additional studies are needed to confirm these trends in antiviral-treatment patients.

Nowadays HBV DNA undetectable and ALT normalisation are the basic requirements with NA treatment. The REVEAL-HBV cohort study in Taiwan showed that increased HBV DNA and ALT levels were predictive for cirrhosis and HCC risk in patients not taking antiviral treatment [15,30,31]. However, in our study, we found no associations between VBT and BBT with cirrhosis or the development of HCC. This discrepancy might reflect key differences between the study populations, whereby the patients in our study were treated with antiviral therapy, compared with no antiviral use in REVEAL-HBV. The predictive value of HBV DNA and ALT levels may be weakened with the application of antiviral therapy.

However, there are also limitations to our study. As this study was a retrospective study based on HIS in a tertiary hospital, aspects such as lifestyle and family history, including HCC family history, smoking, alcohol assumption and diet were not captured in the current study. In addition, there might also be immortal time bias in the study for those patients who did not have serological change because their follow-up period is overestimated in the study.

In conclusion, this study constructed a large antiviral-treated CHB cohort for the analysis of possible risk factors which could affect the development of cirrhosis and HCC. The use of real-world data provided a unique insight into the effect of treatment duration and compliance on clinical outcome. Patients with CHB should be encouraged to adhere to NA therapy in view of the positive impact of good compliance on cirrhosis and HCC risk. Meanwhile, potential protective effects of HBsAg loss on the development of cirrhosis and HCC warrant further investigation. In line with some earlier studies, cirrhosis was still identified as an independent risk factor for HCC in NA-treated patients.
